# Forecasting the Water Demand in Chongqing, China Using a Grey Prediction Model and Recommendations for the Sustainable Development of Urban Water Consumption

**DOI:** 10.3390/ijerph14111386

**Published:** 2017-11-15

**Authors:** Hua’an Wu, Bo Zeng, Meng Zhou

**Affiliations:** 1 College of Rongzhi, Chongqing Technology and Business University, Chongqing 401320, China; wuhuaan@ctbu.edu.cn; 2 Chongqing Key Laboratory of Electronic Commerce & Supply Chain System, Chongqing Technology and Business University, Chongqing 400067, China; mengzhou@email.ctbu.edu.cn

**Keywords:** water demand, grey water forecasting model (GWFM), simulation and prediction, Chongqing economy

## Abstract

High accuracy in water demand predictions is an important basis for the rational allocation of city water resources and forms the basis for sustainable urban development. The shortage of water resources in Chongqing, the youngest central municipality in Southwest China, has significantly increased with the population growth and rapid economic development. In this paper, a new grey water-forecasting model (GWFM) was built based on the data characteristics of water consumption. The parameter estimation and error checking methods of the GWFM model were investigated. Then, the GWFM model was employed to simulate the water demands of Chongqing from 2009 to 2015 and forecast it in 2016. The simulation and prediction errors of the GWFM model was checked, and the results show the GWFM model exhibits better simulation and prediction precisions than those of the classical Grey Model with one variable and single order equation GM(1,1) for short and the frequently-used Discrete Grey Model with one variable and single order equation, DGM(1,1) for short. Finally, the water demand in Chongqing from 2017 to 2022 was forecasted, and some corresponding control measures and recommendations were provided based on the prediction results to ensure a viable water supply and promote the sustainable development of the Chongqing economy.

## 1. Introduction

Water is a basic source of life and a natural and strategic economic resource. The rational utilization of water resources is an important basis for the sustainable development of the economy and society. Chongqing, which is the largest industrial and commercial city in Southwest China, is facing a shortage of water resources.

The average rainfall in the Chongqing area is about 1200 millimetres, which produces about 51.1 billion cubic metres of surface water. The per capita water resource is more than 1640 cubic metres, which is only 33% of the national per capita level and 16% of the world per capita level. The existing water conservancy facilities in Chongqing have a storage capacity of 3.9 billion cubic metres, which is only 7.6% of the total surface water area. The main streams of the three rivers pass through the inland river section of Chongqing. Although Chongqing is rich in water, it can only fetch about 0.6% of surface water annually because of its small flow area, high water intake, and large utilization cost. In 2015, about one million people in the rural areas of Chongqing faced a shortage in the drinking water supply, and the water shortage continued in Western Chongqing. The development and utilization of surface and ground water in Chongqing are insufficient to cope with the rapid urban development, and this means that the absolute amount of water consumption in Chongqing is very large for a long time. At present, the problem of water shortage is attracting significant attention in the engineering field. The shortage of water resources has become an important factor that restricts the sustainable development of Chongqing’s economy, and requires scientific predictions of the water demand to provide targeted control measures.

Presently, many mathematical methods have been employed to forecast the urban water demand [1]. These methods include Artificial Neural Networks (ANNs) [2,3], Fuzzy and neuro-fuzzy models [4,5], the Support Vector Machine [6,7], Markov process [8,9], Moving Window [10], Metaheuristics [11], Regression forecasting model [12], Kalman filter [13], data assimilation technique [14], and System dynamics [15,16]. Previous studies have applied various methods and their hybrids to forecast the water demand. However, the above models were mainly based on Big data, while the sample dates were insufficient to obtain an accurate forecasting result.

The validity and practicability of the grey prediction model have been widely verified in various fields, such as agriculture, industry, society, economy, transportation, energy, and health care [17,18,19,20,21]. We inputted the subject terms ‘grey’ and ‘prediction/forecasting’ in the ‘Web of Science’ using the time span from 2000 to 2016, and 163,553 papers were retrieved. These study findings in the field of grey prediction models mainly focus on parameter optimization [22], objects expansion modelling [23], structure improvement [24], and many other topics. As the first grey prediction model created by Deng [25], the GM(1,1) model [17] has been one of the most important grey models. However, the performance of the raw GM(1,1) model was not stable, and various methods, such as the optimization of the initial and background values [26], smoothness improvement of the raw sequence [27], perfection of the structure and modelling mechanism [28], and combination between grey models and other models [29], have been employed to improve its simulation and prediction accuracy.

In this study, we used the grey system method to build a new prediction model called GWFM for forecasting the water demand in Chongqing, and checked the error of the new model. Then, we used the new model to forecast the water demand between 2017 and 2022 in Chongqing. Finally, we analysed the prediction results, and recommendations and control measures were provided to promote the sustainable development of the Chongqing economy.

The remainder of the paper is organized as follows. In Section 2, we introduce the modelling foundation of the grey prediction model. In Section 3, we establish the GWFM model for forecasting the water demand and deducing its final restored formula. In Section 4, we discuss the method to check the errors of the GWFM model. In Section 5, we use the GWFM model to simulate Chongqing’s water requirement from 2009 to 2015, and forecast it for 2016 using real data. In Section 6, some recommendations are provided. The paper concludes in Section 7.

## 2. The Foundation of Grey Prediction Model: Accumulating Generation Operator (AGO)

For cases with small samples or poor information, the size of the sample is insufficient to find its distribution laws; therefore, the statistical approach cannot be applied to build the predictive models. A grey prediction model (GPM) is a new prediction methodology that focuses on problems involving a small amount of data and poor information. It addresses uncertain systems with partially known information through generating, excavating, and extracting useful information from what is available. The theory enables a correct description of a system’s running behaviour and its evolution law, and thus generates quantitative predictions of future system changes. Incomplete and inaccurate information is the basic characteristic of uncertainty systems [17].

The Grey prediction model (GPM) identifies the change laws of uncertain systems by mining and organizing the limited available original data, which is called grey sequence generation. The accumulating and inverse accumulating generation operators are two important methods of the grey sequence generation and play extremely important roles in GPM [30], which are defined as follows.

**Definition 1**. *Assumes that* $W^{\left(0\right)}=\left(w^{\left(0\right)}\left(1\right),w^{\left(0\right)}\left(2\right),\cdots ,w^{\left(0\right)}\left(n\right)\right)$ *is an original sequence, and the symbol* D *represents a kind of mathematical operational method. When* D *is applied once on* $W^{\left(0\right)}$*, we obtain the following:*

$$W^{\left(0\right)}D^{\left(1\right)}=W^{\left(1\right)}=(w^{\left(1\right)}\left(1\right),w^{\left(1\right)}\left(2\right),\cdots ,w^{\left(1\right)}\left(n\right)),$$

where

$$w^{\left(1\right)}\left(k\right)=\sum_{i=1}^{k}w^{\left(0\right)}\left(i\right),k=1,2,\cdots n$$

Then, D is called the first-order accumulating generation operator of $w^{\left(0\right)}$ [17], and is denoted by 1-AGO. If D is applied r times on $W^{\left(0\right)}$, we obtain the following:

$$W^{\left(0\right)}D^{\left(r\right)}=W^{\left(r\right)}=(w^{\left(r\right)}\left(1\right),w^{\left(r\right)}\left(2\right),\cdots ,w^{\left(r\right)}\left(n\right)),$$

where

$$w^{\left(r\right)}\left(k\right)=\sum_{i=1}^{k}w^{\left(r-1\right)}\left(i\right),k=1,2,\cdots n\;\mathrm{and}\;r\in Z^{+}$$

Then, D is called the *r*-order accumulating generation operator of $X^{\left(0\right)}$, and is denoted by *r*-AGO.

**Figure 1:**
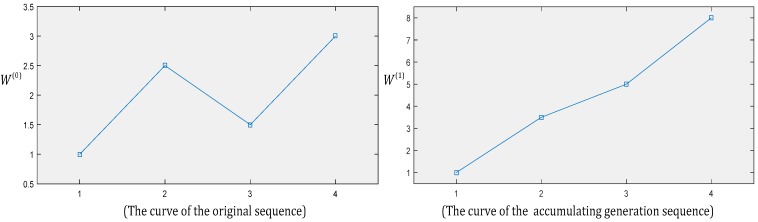
Curves with the raw sequence and its accumulating generation sequence.

The accumulating generation operator D can weaken the randomness of original data. For example, for a raw sequence where $X^{\left(0\right)}=\left(1,2.5,1.5,3\right)$, its 1-AGO sequence is $X^{\left(1\right)}=\left(1,3.5,5,8\right)$. The curves of these sequences are illustrated in Figure 1. When comparing the two curves, it is clear that the trend of sequence $X^{\left(1\right)}$ is more obvious than that of sequence $X^{\left(0\right)}$ in Figure 1.

Accumulating generation operation (AGO) is a method employed to whitenize a grey process. It plays an extremely important role in grey system theory. Through AGO, one can potentially uncover a development tendency existing in the process of accumulating grey quantities so that the characteristics and laws of integration hidden in the chaotic original data can be sufficiently revealed.

Urban water demand is affected by a series of uncertain factors, such as the population, economic development, and social conditions. Meanwhile, the statistical data on the urban water demand in Chongqing is very limited since it became a municipality directly under the central government in 1997. Therefore, the traditional statistical methods based on large sample data are difficult to apply when simulating and forecasting the urban water demand. 

Therefore, we will choose the grey prediction model to study the prediction issue of urban water demand in Chongqing in this paper.

## 3. Grey System Model for Forecasting of Water Demand

**Definition** **2.** *[17] assumes that* $W^{\left(0\right)}$*,* $W^{\left(1\right)}$ *are given by Definition 1, and* $Z^{\left(1\right)}$ *denotes the immediate mean generation sequence of* $W^{\left(1\right)}$ *as follows:*

$$Z^{\left(1\right)}=\left(z^{\left(1\right)}\left(2\right),z^{\left(1\right)}\left(3\right),\cdots ,z^{\left(1\right)}\left(n\right)\right)$$

where $z^{\left(1\right)}\left(t\right)=0.5\times \left[w^{\left(1\right)}\left(t\right)+w^{\left(1\right)}\left(t-1\right)\right]$, t=2,3⋯,n. Then,

(1) $$w^{\left(0\right)}\left(t\right)+az^{\left(1\right)}\left(t\right)=0.5\left(2t-1\right)b+c$$

is the basic form of the water demand prediction model.

The basic form of traditional grey prediction GM(1,1) is $x^{\left(0\right)}\left(t\right)+az^{\left(1\right)}\left(t\right)=b$, and its final time response function is ${\hat{x}}^{\left(0\right)}\left(t+1\right)=\left(1-e^{a}\right)\left(x^{\left(0\right)}\left(1\right)-b/a\right)e^{-at}$, which can only simulate a homogenous exponential sequence. However, in the real world, the sequence with the characteristic of homogeneous exponential growth is only a special case in the ideal state; more systematic behavior sequences exhibit approximately non-homogeneous exponential growth characteristics. In this case, the performance of the traditional grey prediction model based on $x^{\left(0\right)}\left(t\right)+az^{\left(1\right)}\left(t\right)=b$ is poor. Since the proposed model, Equation (1), has a more complete structure and can achieve unbiased simulations and predictions for both homogenous and non-homogenous exponential sequences and linear functions, it can be seen that Equation (1) exhibits a better performance than that of traditional grey prediction models.

According to the inverse process of accumulating generation operator in Definition 1 [17],

(2) $$w^{\left(0\right)}\left(t\right)=w^{\left(1\right)}\left(t\right)-w^{\left(1\right)}\left(t-1\right),t=2,3,\cdots n$$

Substituting $z^{\left(1\right)}\left(t\right)=0.5\times \left[w^{\left(1\right)}\left(t\right)+w^{\left(1\right)}\left(t-1\right)\right]$ and Equation (2) into Equation (1) when t=2,3,⋯n, we can obtain the following equation:

$$w^{\left(1\right)}\left(t\right)-w^{\left(1\right)}\left(t-1\right)+0.5aw^{\left(1\right)}\left(t\right)+0.5aw^{\left(1\right)}\left(t-1\right)=0.5\left(2t-1\right)b+c$$

that is

$$\left(1+0.5a\right)w^{\left(1\right)}\left(t\right)-(1-0.5a)w^{\left(1\right)}\left(t-1\right)=0.5\left(2t-1\right)b+c$$

(3) $$w^{\left(1\right)}\left(t\right)=\frac{1-0.5a}{1+0.5a}w^{\left(1\right)}\left(t-1\right)+\frac{b}{1+0.5a}t-\frac{0.5b-c}{1+0.5a}$$

Let

$$\delta_{1}=\frac{1-0.5a}{1+0.5a},\delta_{2}=\frac{b}{1+0.5a},\delta_{3}=\frac{0.5b-c}{1+0.5a}$$

Then, Equation (3) can be transformed as follows:

(4) $$w^{\left(1\right)}\left(t\right)=\delta_{1}w^{\left(1\right)}\left(t-1\right)+\delta_{2}t+\delta_{3},\;t=2,3,\cdots ,n$$

Assume that ${\hat{x}}^{\left(1\right)}\left(k\right)$ is the simulation of $x^{\left(1\right)}\left(k\right)$. To minimise the simulation error $x^{\left(1\right)}\left(k\right)$, the following condition needs to be satisfied:

$$S=\min{\sum_{t=2}^{n}\left[w^{\left(1\right)}\left(t\right)-{\hat{w}}^{\left(1\right)}\left(t\right)\right]}^{2}=\min{\sum_{k=2}^{n}\left[w^{\left(1\right)}\left(t\right)-{\hat{\delta }}_{1}w^{\left(1\right)}\left(t-1\right)-{\hat{\delta }}_{2}t-{\hat{\delta }}_{3}\right]}^{2}$$

According to OLS (Ordinary Least Square), we minimise S with respect to parameters $\delta_{1},\delta_{2},\delta_{3}$ to obtain the following equation:

$$\left\{ \begin{array}{c} \frac{\partial S}{\partial {\hat{\delta }}_{1}}=-2\sum_{t=2}^{n}\left[w^{\left(1\right)}\left(t\right)-{\hat{\delta }}_{1}w^{\left(1\right)}\left(t-1\right)-{\hat{\delta }}_{2}t-{\hat{\delta }}_{3}\right]w^{\left(1\right)}\left(t-1\right)=0\\ \frac{\partial S}{\partial {\hat{\delta }}_{2}}=-2\sum_{t=2}^{n}\left[w^{\left(1\right)}\left(t\right)-{\hat{\delta }}_{1}w^{\left(1\right)}\left(t-1\right)-{\hat{\delta }}_{2}t-{\hat{\delta }}_{3}\right]t=0\\ \frac{\partial S}{\partial {\hat{\delta }}_{3}}=-2\sum_{t=2}^{n}\left[w^{\left(1\right)}\left(t\right)-{\hat{\delta }}_{1}w^{\left(1\right)}\left(t-1\right)-{\hat{\delta }}_{2}t-{\hat{\delta }}_{3}\right]=0 \end{array}\right..$$

Then,

(5) $$\left\{ \begin{array}{c} {\hat{\delta }}_{1}\sum_{t=2}^{n}{\left[w^{\left(1\right)}\left(t-1\right)\right]}^{2}+{\hat{\delta }}_{2}\sum_{t=2}^{n}\left[tw^{\left(1\right)}\left(t-1\right)\right]+{\hat{\delta }}_{3}\sum_{t=2}^{n}w^{\left(1\right)}\left(t-1\right)\\ \;=\sum_{t=2}^{n}\left[w^{\left(1\right)}\left(t\right)w^{\left(1\right)}\left(t-1\right)\right]\\ {\hat{\delta }}_{1}\sum_{t=2}^{n}tw^{\left(1\right)}\left(t-1\right)+{\hat{\delta }}_{2}\sum_{t=2}^{n}t^{2}+{\hat{\delta }}_{3}\sum_{t=2}^{n}t=\sum_{t=2}^{n}tw^{\left(1\right)}\left(t\right)\\ {\hat{\delta }}_{1}\sum_{t=2}^{n}w^{\left(1\right)}\left(t-1\right)+{\hat{\delta }}_{2}\sum_{t=2}^{n}k+{\hat{\delta }}_{3}(n-1)=\sum_{t=2}^{n}w^{\left(1\right)}\left(t\right) \end{array}\right..$$

Next, the calculation of the unknown parameters ${\hat{\delta }}_{1},{\hat{\delta }}_{2},{\hat{\delta }}_{3}$ in Equation (5) is presented. According to Cramer’s rule, we can obtain the following results:

$$D=\left|\begin{array}{ccc} \sum_{t=2}^{n}{\left[w^{\left(1\right)}\left(t-1\right)\right]}^{2} & \sum_{t=2}^{n}kw^{\left(1\right)}\left(t-1\right) & \sum_{t=2}^{n}w^{\left(1\right)}\left(t-1\right)\\ \sum_{t=2}^{n}tw^{\left(1\right)}\left(t-1\right) & \sum_{t=2}^{n}t^{2} & \sum_{t=2}^{n}t\\ \sum_{t=2}^{n}w^{\left(1\right)}\left(t-1\right) & \sum_{t=2}^{n}t & n-1 \end{array}\right|$$

$$D_{1}=\left|\begin{array}{ccc} \sum_{t=2}^{n}\left[w^{\left(1\right)}\left(t\right)w^{\left(1\right)}\left(t-1\right)\right] & \sum_{t=2}^{n}kw^{\left(1\right)}\left(t-1\right) & \sum_{t=2}^{n}w^{\left(1\right)}\left(t-1\right)\\ \sum_{t=2}^{n}tw^{\left(1\right)}\left(t\right) & \sum_{t=2}^{n}t^{2} & \sum_{t=2}^{n}t\\ \sum_{t=2}^{n}w^{\left(1\right)}\left(t\right) & \sum_{t=2}^{n}t & n-1 \end{array}\right|$$

$$D_{2}=\left|\begin{array}{ccc} \sum_{t=2}^{n}{\left[w^{\left(1\right)}\left(t-1\right)\right]}^{2} & \sum_{t=2}^{n}\left[w^{\left(1\right)}\left(t\right)w^{\left(1\right)}\left(t-1\right)\right] & \sum_{t=2}^{n}w^{\left(1\right)}\left(t-1\right)\\ \sum_{t=2}^{n}tw^{\left(1\right)}\left(t-1\right) & \sum_{t=2}^{n}tw^{\left(1\right)}\left(t\right) & \sum_{t=2}^{n}t\\ \sum_{t=2}^{n}w^{\left(1\right)}\left(t-1\right) & \sum_{t=2}^{n}w^{\left(1\right)}\left(t\right) & n-1 \end{array}\right|$$

$$D_{3}=\left|\begin{array}{ccc} \sum_{t=2}^{n}{\left[w^{\left(1\right)}\left(t-1\right)\right]}^{2} & \sum_{t=2}^{n}tw^{\left(1\right)}\left(t-1\right) & \sum_{t=2}^{n}\left[w^{\left(1\right)}\left(t\right)w^{\left(1\right)}\left(t-1\right)\right]\\ \sum_{t=2}^{n}tw^{\left(1\right)}\left(t-1\right) & \sum_{t=2}^{n}t^{2} & \sum_{t=2}^{n}tw^{\left(1\right)}\left(t\right)\\ \sum_{t=2}^{n}w^{\left(1\right)}\left(t-1\right) & \sum_{t=2}^{n}t & \sum_{t=2}^{n}w^{\left(1\right)}\left(t\right) \end{array}\right|$$

If D≠0, based on Cramer’s rule, the parameters ${\hat{\delta }}_{1},{\hat{\delta }}_{2},{\hat{\delta }}_{3}$ can be calculated as follows:

$${\hat{\delta }}_{1}=\frac{D_{1}}{D},{\hat{\delta }}_{2}=\frac{D_{2}}{D},{\hat{\delta }}_{3}=\frac{D_{3}}{D}$$

Since

$${\hat{\delta }}_{1}=\frac{1-0.5\hat{a}}{1+0.5\hat{a}},{\hat{\delta }}_{2}=\frac{\hat{b}}{1+0.5\hat{a}},{\hat{\delta }}_{3}=\frac{0.5\hat{b}-\hat{c}}{1+0.5\hat{a}},$$

the parameters *a*, *b*, and *c* can be obtained as follows:

$$\hat{a}=\frac{2(1-{\hat{\delta }}_{1})}{1+{\hat{\delta }}_{1}},\hat{b}=\frac{2{\hat{\delta }}_{2}}{1+{\hat{\delta }}_{1}},\hat{c}=\frac{2{\hat{\delta }}_{2}-4{\hat{\delta }}_{3}}{1+{\hat{\delta }}_{1}}$$

From Equation (3),

(6) $${\hat{w}}^{\left(1\right)}\left(t\right)=\frac{1-0.5\hat{a}}{1+0.5\hat{a}}{\hat{w}}^{\left(1\right)}\left(t-1\right)+\frac{\hat{b}}{1+0.5\hat{a}}t-\frac{0.5\hat{b}-\hat{c}}{1+0.5\hat{a}}$$

and the restored expression is as ${\hat{w}}^{\left(0\right)}\left(t\right)={\hat{w}}^{\left(1\right)}\left(t\right)-{\hat{w}}^{\left(1\right)}\left(t-1\right)$.

In the grey prediction model, we often applied $w^{\left(0\right)}\left(1\right)=w^{\left(1\right)}\left(1\right)={\hat{w}}^{\left(1\right)}\left(1\right)$ as the initial value to deduce the time-response function of ${\hat{w}}^{\left(1\right)}\left(t\right)$ in Equation (6). The detailed deduction process is as follows:

when t=2,

$${\hat{w}}^{\left(1\right)}\left(2\right)=\left(\frac{1-0.5\hat{a}}{1+0.5\hat{a}}\right)w^{\left(0\right)}\left(1\right)+\left(\frac{2\hat{b}}{1+0.5\hat{a}}-\frac{0.5\hat{b}-\hat{c}}{1+0.5\hat{a}}\right)$$

when t=3,

$$\begin{array}{c} {\hat{w}}^{\left(1\right)}\left(3\right)={\left(\frac{1-0.5\hat{a}}{1+0.5\hat{a}}\right)}^{2}w^{\left(0\right)}\left(1\right)+\frac{1-0.5\hat{a}}{1+0.5\hat{a}}\left(\frac{2\hat{b}}{1+0.5\hat{a}}+\frac{1-0.5\hat{a}}{1+0.5\hat{a}}\right)+\left(\frac{3\hat{b}}{1+0.5\hat{a}}+\frac{0.5\hat{b}-\hat{c}}{1+0.5\hat{a}}\right)\\ \vdots \end{array}$$

when t=k,

(7) $$\begin{array}{l} {\hat{w}}^{\left(1\right)}\left(k\right)=w^{\left(0\right)}\left(1\right){\left(\frac{1-0.5\hat{a}}{1+0.5\hat{a}}\right)}^{k-1}+\left(\frac{2\cdot 2\hat{b}}{1+0.5\hat{a}}+\frac{0.5\hat{b}-\hat{c}}{1+0.5\hat{a}}\right){\left(\frac{1-0.5\hat{a}}{1+0.5\hat{a}}\right)}^{k-2}+↘\\ \;\;\;\;\;\;\;\;\;\;\;\;\;↖\;\;\left(\frac{3\cdot 2\hat{b}}{1+0.5\hat{a}}+\frac{0.5\hat{b}-\hat{c}}{1+0.5\hat{a}}\right){\left(\frac{1-0.5\hat{a}}{1+0.5\hat{a}}\right)}^{k-3}+\cdots +\left(\frac{k\cdot 2\hat{b}}{1+0.5\hat{a}}+\frac{0.5\hat{b}-\hat{c}}{1+0.5\hat{a}}\right)\left(\frac{1-0.5\hat{a}}{1+0.5\hat{a}}\right) \end{array}$$

Arranging Equation (7), we obtain the following equation:

(8) $${\hat{w}}^{\left(1\right)}\left(k\right)=w^{\left(0\right)}\left(1\right){\left(\frac{1-0.5\hat{a}}{1+0.5\hat{a}}\right)}^{k-1}+\sum_{i=0}^{k-2}\left[\left(k-i\right)\frac{\hat{b}}{1+0.5\hat{a}}+\frac{0.5\hat{b}-\hat{c}}{1+0.5\hat{a}}\right]{\left(\frac{1-0.5\hat{a}}{1+0.5\hat{a}}\right)}^{i}$$

Equation (8) can be simplified as follows:

(9) $${\hat{w}}^{\left(1\right)}\left(k\right)=w^{\left(0\right)}\left(1\right){\hat{\delta }}_{1}^{k-1}+\sum_{i=0}^{k-2}\left[\left(k-i\right){\hat{\delta }}_{2}+{\hat{\delta }}_{3}\right]{\hat{\delta }}_{1}^{i}$$

From Equation (9), when k=2,3,⋯, the final expression of ${\hat{w}}^{\left(0\right)}\left(k\right)={\hat{w}}^{\left(1\right)}\left(k\right)-{\hat{w}}^{\left(1\right)}\left(k-1\right)$ is as follows:

(10) $${\hat{w}}^{\left(0\right)}\left(k\right)=\left[w^{\left(0\right)}\left(1\right)\left({\hat{\delta }}_{1}-1\right)+\left(2{\hat{\delta }}_{2}+{\hat{\delta }}_{3}\right)\right]{\hat{\delta }}_{1}^{k-2}+\sum_{i=0}^{k-3}{\hat{\delta }}_{2}{\hat{\delta }}_{1}^{i}$$

Equation (10) is called the GWFM model. Compared with the classical GM(1,1) model, the proposed GWFM model can unbiasedly simulate a homogeneous exponential sequence, a non-homogeneous exponential sequence, and a linear function sequence (detailed proofs are presented elsewhere), which demonstrates its good performance.

## 4. Method to Check for the GWFM Model Error

One model’s performance includes two aspects: simulation performance and prediction performance. Normally, only the models that pass various tests can be meaningfully employed to make predictions.

**Definition** **3.** *Assumes that a raw sequence is* $W^{\left(0\right)}⊗$*, as follows:*

$$W^{\left(0\right)}=\left(w^{\left(0\right)}\left(1\right),w^{\left(0\right)}\left(2\right),\cdots ,w^{\left(0\right)}\left(n\right),w^{\left(0\right)}\left(n+1\right),\cdots ,w^{\left(0\right)}\left(n+t\right)\right)$$

A subsequence which is composed of the first n elements of the sequence $W^{\left(0\right)}$ is used to build the GWFM model and the simulation sequence is ${\hat{S}}^{\left(0\right)}$, as follows:

$${\hat{S}}^{\left(0\right)}=\left({\hat{w}}^{\left(0\right)}\left(1\right),{\hat{w}}^{\left(0\right)}\left(2\right),\cdots ,{\hat{w}}^{\left(0\right)}\left(n\right)\right)$$

We use the GWFM model to forecast the latter p-step data, and the prediction sequence is ${\hat{F}}^{\left(0\right)}$, as follows:

$${\hat{F}}^{\left(0\right)}=\left({\hat{w}}^{\left(0\right)}\left(n+1\right),{\hat{w}}^{\left(0\right)}\left(n+2\right),\cdots ,{\hat{w}}^{\left(0\right)}\left(n+t\right)\right)$$

The error sequences of ${\hat{S}}^{\left(0\right)}$ and ${\hat{F}}^{\left(0\right)}$ are $\varepsilon_{s}$ and $\varepsilon_{F}$, respectively, as follows:

$$\varepsilon_{S}=\left(\varepsilon_{S}\left(1\right),\varepsilon_{S}\left(2\right),\cdots ,\varepsilon_{S}\left(n\right)\right)$$

$$\varepsilon_{F}=\left(\varepsilon_{F}\left(n+1\right),\varepsilon_{F}\left(n+2\right),\cdots ,\varepsilon_{F}\left(n+t\right)\right)$$

where

$$\varepsilon_{S}\left(u\right)=w^{\left(0\right)}\left(u\right)-{\hat{w}}^{\left(0\right)}\left(u\right),u=1,2,\cdots ,n$$

$$\varepsilon_{F}\left(v\right)=w^{\left(0\right)}\left(v\right)-{\hat{w}}^{\left(0\right)}\left(v\right),v=n+1,n+2,\cdots ,n+t$$

The relative simulation percentage error (RSPE) of simulation sequence ${\hat{S}}^{\left(0\right)}$ is $\Delta_{S}$, as follows:

$$\Delta_{S}=\left(\Delta_{S}\left(1\right),\Delta_{S}\left(2\right),\cdots ,\Delta_{S}\left(n\right)\right)$$

where

$$\Delta_{S}\left(u\right)=\left|\frac{\varepsilon_{S}\left(u\right)}{w^{\left(0\right)}\left(u\right)}\times 100\%\right|,u=1,2,\cdots ,n$$

The mean relative simulation percentage error (MRSPE) of simulation sequence is ${\bar{\Delta }}_{S}$, as follows:

$${\bar{\Delta }}_{S}=\frac{1}{n}\sum_{u=1}^{n}\Delta_{S}\left(u\right)$$

The relative prediction percentage error (RFPE) of forecast sequence is $\Delta_{F}$, as follows:

$$\Delta_{F}=\left(\Delta_{F}\left(n+1\right),\Delta_{F}\left(n+2\right),\cdots ,\Delta_{F}\left(n+t\right)\right)$$

where

$$\Delta_{F}\left(v\right)=\left|\frac{\varepsilon_{F}\left(v\right)}{w^{\left(0\right)}\left(v\right)}\times 100\%\right|,v=n+1,n+2,\cdots ,n+t$$

The mean relative prediction percentage error (MRPPE) of prediction sequence is ${\bar{\Delta }}_{F}$, as follows:

$${\bar{\Delta }}_{F}=\frac{1}{t}\sum_{v=n+1}^{n+t}\Delta_{F}\left(v\right)$$

The comprehensive mean relative percentage error (CMRPE) of the GWFM mode is Δ, as follows:

$$\Delta =\frac{{\bar{\Delta }}_{S}+{\bar{\Delta }}_{F}}{2}$$

For giving threshold values α and β (the threshold is set according to the specific situation of the system), when ${\bar{\Delta }}_{S}<\alpha$ and ${\bar{\Delta }}_{F}<\beta$ hold true, the GWFM model is said to be error-satisfactory. However, when the size of the modeling data is small, the original sequence cannot be divided into “simulation subsequence” and “prediction subsequence”. At this time, we only test the simulation error of the model, and the test of prediction error will be omitted.

## 5. Forecasting the Water Demand in Chongqing Using GWFM

Urban water consumption is the gross water consumption of users, including water loss during transport. It comprises live water, manufactured water, and ecological environment supplemental water. The water consumption in Chongqing from 2009 to 2016 is presented in Table 1.

In Section 4, we have discussed that a model can only be used to forecast when the simulation and prediction performances of the model have passed the relevant tests. Hence, in this section, the data from 2009 to 2015 will be used to build the GWFM model of the Chongqing water demand. The remaining data will be employed to check the prediction performance of the GWFM model.

Table 1 shows the original sequence of $W^{\left(0\right)}$, as follows:

$$\begin{array}{l} W^{\left(0\right)}=\left(w^{\left(0\right)}\left(1\right),w^{\left(0\right)}\left(2\right),w^{\left(0\right)}\left(3\right),w^{\left(0\right)}\left(4\right),w^{\left(0\right)}5,w^{\left(0\right)}\left(6\right)\right)\\ \;\;\;\;\;\;\;=\left(85.3032,86.3866,86.7976,82.9360,83.9066,80.4687,78.9802\right) \end{array}$$

Then, we established the GWFM model of sequence $W^{\left(0\right)}$ and wrote the MATLAB program to calculate the parameters $\delta_{1},\delta_{2},\delta_{3}$ and a,b,c. The results are shown in Table 2.

We can build the GWFM model according to Equation (10), which can be used to forecast the water demand in Chongqing, as follows:

$${\hat{w}}^{\left(0\right)}\left(k\right)=\left[w^{\left(0\right)}\left(1\right)\left({\hat{\delta }}_{1}-1\right)+\left(2{\hat{\delta }}_{2}+{\hat{\delta }}_{3}\right)\right]{\hat{\delta }}_{1}^{k-2}+\sum_{i=0}^{k-3}{\hat{\delta }}_{2}{\hat{\delta }}_{1}^{i}$$

Substituting parameters in Table 2 into Equation (10), we can obtain:

(11) $${\hat{w}}^{\left(0\right)}\left(t\right)=87.04524\times 1.05899^{t-2}-6.53865\sum_{i=0}^{t-3}1.05899^{i}$$

In Equation (11), when t=2,3,⋯,n, ${\hat{w}}^{\left(0\right)}\left(t\right)$ is called simulation data; and when t=n+1,n+2,⋯, ${\hat{w}}^{\left(0\right)}\left(t\right)$ is called prediction data. Hence, we can calculate the simulation data ${\hat{w}}^{\left(0\right)}\left(t\right)$, the simulation residual error ε(t), the relative simulation percentage error Δ(t), and the mean relative simulation percentage error $\bar{\Delta }$. To compare the simulation and prediction performances of the GWFM, the classic GM(1,1) model and the frequently-used DGM(1,1) model were also applied to simulate and forecast the water demand in Chongqing with the same data. All simulation and prediction results of the above three models are shown in Table 3. Besides, the parameter values of the GM(1,1) and DGM(1,1) models are shown in Table 4, as follows.

Table 3 shows that the GWFM model has the best comprehensive mean relative percentage error (CMRPE) among the above three models, closely followed by the DGM(1,1) and GM(1,1) models. The grey model error level reference table [17] shows that the comprehensive grade of the GWFM model is Grade I, which can be used for predictions. We used the GWFM model to forecast the water demand in Chongqing from 2017 to 2022, and the results are shown in Table 5.

Table 5 shows that the water demand in Chongqing had a slow downward pattern from 2017 to 2022. This is mainly because of the strict water management system introduced by Chongqing, which resulted in the following benefits: an annual decrease of the water consumption per million yuan of industrial added value, significant improvement in the effective utilization coefficient of farmland irrigation water, and significant improvement of the water quality in the water areas of important rivers and lakes in the Chongqing area.

## 6. Recommendations for Sustainable Development of Urban Water Consumption in Chongqing

As seen in Table 5, the absolute quantity of the water demand is still very large. Therefore, some recommendations should be provided to ensure the effective supply of water resources to promote the sustainable development of Chongqing’s economy and society. 

Firstly, the economic development scale and configuration of a reasonable water resource should be adjusted. The economic development scale, speed, and pattern must be adjusted according to the water-resource bearing capacity and rational configuration of Chongqing. The water resource, population, cultivated land, and mineral exploitation must be configured for optimal benefit. In the drought areas, urban development and scale must be limited. Water project construction must be accelerated based on a rational plan, and the optimal allocation of a water resources system must be implemented. 

Secondly, the utilization efficiency of water resources in Chongqing should be improved. Chongqing is an industrial city dominated by manufacturing and the industrial water consumption is an important part of Chongqing water consumption. Hence, reducing the water consumption of industrial added value of ten thousand yuan in Chongqing is a key measure for controlling the water consumption scale in Chongqing. Besides, the water-saving consciousness of Chongqing citizens should be cultivated, and it can employ the appropriate use of price leverage to strive for water conservation.

Thirdly, a strict water management system and unified water resources management operation mechanism should be established. The solution to the water problem, which involves all aspects of nature and human society, involves huge system engineering. Water saving, efficient water consumption, unified management, and the optimal allocation of water resources form the core of system engineering to relieve the contradiction between the supply and demand of water resources. A public-management function must be established, and using the market economy, the development and utilization of water resources must be controlled. Furthermore, a water resources management operation mechanism must be formulated to promote the utilization and protection of sustainable water environments.

## 7. Conclusions

Scientific and accurate forecasting of urban water demand is the basis of the sustainable development of urban water consumption. Chongqing is an urban area facing a shortage of water resources. Recently, the rapid economic development and population growth highlighted the contradiction between the supply and demand of water resources. In this study, we built a GWFM model of urban water demand based on the grey system model, deduced the parameter estimation, and eventually restored the formula and checked the model error testing method. In summary, the GWFM model successfully forecasted the urban water demand in Chongqing from 2017 to 2025, and corresponding control measures and recommendations were provided based on the forecasting result.

## Figures and Tables

**Table 1:** Water consumption in Chongqing from 2009 to 2016 (Unit: hundred million cubic metres).

Year	2009	2010	2011	2012	2013	2014	2015	2016
k	k=1	k=2	k=3	k=4	k=5	k=6	k=7	k=8
Water consumption	85.3032	86.3866	86.7976	82.9360	83.9066	80.4687	78.9802	77.4800

Source: Chongqing water resource communique from 2009 to 2016 [31].

**Table 2:** Value of the parameters of the GWFM model with sequence $W^{\left(0\right)}$.

Parameter	${\hat{\delta }}_{1}$	${\hat{\delta }}_{2}$	${\hat{\delta }}_{3}$	$\hat{a}$	$\hat{b}$	$\hat{c}$
Value	1.05899	−6.538648	95.09049	−0.05730	−6.351328	−183.70365

**Table 3:** Simulation/forecasted values and errors of GFWM, DGM(1,1), and GM(1,1).

Serial Number k	Raw Data $w^{\left(0\right)}\left(t\right)$	Model GFWM			Model DGM(1,1)			Model GM(1,1)		
		${\hat{w}}^{\left(0\right)}\left(t\right)$	$\varepsilon_{S}\left(k\right)$	$\Delta_{S}\left(k\right)$	${\hat{w}}^{\left(0\right)}\left(t\right)$	$\varepsilon_{S}\left(k\right)$	$\Delta_{S}\left(k\right)$	${\hat{w}}^{\left(0\right)}\left(t\right)$	$\varepsilon_{S}\left(k\right)$	$\Delta_{S}\left(k\right)$
**In-sample**										
**1**	85.3032	85.3032	0.000	0.000%	85.3032	0.000	0.000%	85.3032	0.000	0.000%
**2**	86.3866	87.0452	−0.659	0.763%	85.3032	0.831	0.962%	87.2096	−0.823	0.953%
**3**	86.7976	85.6414	1.156	1.332%	87.2171	−1.209	1.393%	85.5835	1.214	1.399%
**4**	82.9360	84.1548	−1.219	1.470%	85.5888	1.055	1.272%	83.9877	−1.052	1.268%
**5**	83.9066	82.5804	1.326	1.580%	83.9908	−1.484	1.769%	82.4216	1.485	1.770%
**6**	80.4687	80.9132	−0.445	0.553%	82.4227	0.415	0.516%	80.8847	−0.416	0.517%
**7**	78.9802	79.1476	−0.167	0.211%	80.8839	0.394	0.499%	79.3765	−0.396	0.502%
**Out-of-sample**										
**8**	77.4800	77.2784	0.202	0.260%	77.8919	0.412	0.532%	77.8965	−0.417	0.539%
**CMRPE (**Δ**)**		0.621%			0.802%			0.804%		

**Table 4:** Values of the parameters of the DGM(1,1) model and GM(1,1).

Model	DGM(1,1)	GM(1,1)
Parameter value	$\beta_{1}=0.98133$; $\beta_{2}=0.98133$	a=0.01882; b=89.63857

**Table 5:** Prediction data of the water demand in Chongqing from 2017 to 2022.

Year	2017	2018	2019	2020	2021	2022
Water demand	75.298	73.201	70.981	68.629	66.139	63.502

Unit: hundred million cubic metres.
